# Effect of Vacuum Sealed Drainage on Recovery of Gastrointestinal Function in Gastric Cancer Patients after Radical Gastrectomy

**DOI:** 10.7497/j.issn.2095-3941.2012.04.008

**Published:** 2012-12

**Authors:** Yan Wang, Hui-ying Wang, Chun-mei Wang

**Affiliations:** 1 Department of Gastroenterological Cancer, Tianjin Medical University Cancer Institute and Hospital, Tianjin 300060, China.; 2 Nursing School, Tianjin Medical University, Tianjin 300070, China.

**Keywords:** drainage, gastrectomy, recovery

## Abstract

**Objective:**

This study aims to explore the effect of vacuum sealed drainage on the recovery of gastrointestinal function in gastric patients after radical gastrectomy.

**Methods:**

One hundred and twenty patients who received radical for gastric cancer were randomly divided into two groups. Patients in the control group received continuous gastrointestinal decompression to drain the gastric juices after radical gastrectomy, whereas patients in the treatment group received vacuum sealed drainage. The postoperative variables between the two groups were compared, including time of bowel sound reoccurrence, time of the first flatus, indwelling time of gastric tube, days of hospitalization, and complications, such as anastomotic leakage, intestinal obstruction, wound infection, pulmonary infection, fever, and pharyngitis. SPSS 13.0 was used to analyze the data.

**Results:**

Significant differences in the following variables were observed in patients between the two groups: time of bowel sound reoccurrence, time of the first flatus, indwelling time of gastric tube, and length of hospitalization of the patients. The value of each of these variables was much smaller in the treatment group than in the control group (*P*<0.05). No significant difference was found in the incidence of anastomotic leakage, intestinal obstruction, and wound infection among patients between the two groups (*P*>0.05). However, a significant differences were observed in the incidence of pulmonary infection, fever, and pharyngitis among the patients between the two groups (*P*<0.05), with much lower incidence of the variables in the treatment group than in the control group.

**Conclusions:**

Vacuum sealed drainage used in gastric cancer patients after radical gastrectomy can accelerate the recovery of gastrointestinal function and reduce postoperative complications. Moreover, it shortens the indwelling time of the gastric tube, thereby making the patients feel comfortable without the disturbance from the gastric tube.

## Introduction

Before 2002, gastrointestinal decompression has been a common treatment for patients after radical gastrectomy^[^^1^^]^. According to some studies, applying gastrointestinal decompression after operation could drain away air and gastric contents in the stomach and intestines through a nasogastric tube, consequently reducing pressure, bloating, and abdominal distension, thereby, promoting the recovery of gastrointestinal function^[^^2^^]^. However, some studies^[^^3^^–^^7^^]^ have shown that continuous decompression has several deficiencies, and does not decrease the incidence of postoperative complications. Carrere^[^^8^^]^ found through a randomized controlled trial that routine preventive decompression after abdominal surgery is unnecessary. However, further research is needed to verify this finding. One viewpoint at present believes that continuous gastrointestinal decompression may cause much loss of digestive juices, and delay the recovery of gastrointestinal function ^[^^9^^]^.

The vacuum sealed drainage being used recently has been derived from the concept of rapid recovery, which drains out the gastric juices by natural gravity instead of continuous gastrointestinal decompression. Studies have demonstrated that gastric retention can be observed when the nasogastric tube is unclipped intermittently to suck gastric fluid with vacuum aspiration. These studies have also found that vacuum sealed drainage causes lower incidence of postoperative complications compared with continuous decompression^[^^10^^]^. Although the use of vacuum sealed drainage has become a routine practice in some foreign hospitals, continuous gastrointestinal decompression is still being used in China. Clinical practice has also proven that vacuum sealed drainage is simple, and minimizes the workload of nurses. The present study compares the effects of vacuum sealed drainage and continuous gastrointestinal decompression on the recovery of gastrointestinal function and the incidence of complications among gastric cancer patients after radical gastrectomy.

## Patients and Methods

### Patients

A total of 185 patients with gastric cancer underwent radical gastrectomy in our hospital between May 2010 and May 2011. In total, 120 patients were selected and fully informed about the objectives and methods of this study. Informed consent was obtained from each participant. The study was approved by the institute’s ethical committee. The patients were randomly divided into two groups using a random number table. The control group was also composed of 60 patients, of which 36 were male and 24 were female, with an average age of 59.24±8.26. The numbers of the patients with stage I, II, III, IV were 8, 15, 31, 6, respectively. Forty three of the patients in the control group had distal subtotal gastrectomy, whereas 17 had proximate gastrectomy. Forty two of the patients in the control group received enteral nutrition. The treatment group was composed of 60 patients, of which 41 were male and 19 were female, with an average age of 57.93±8.95. The numbers of the patients with stage I, II, III, IV were 7, 16, 32, 5, respectively. Forty of the patients in the treatment group had distal subtotal gastrectomy, whereas 20 had proximate gastrectomy. Enteral nutrition was provided to 39 of the patients in the treatment group. Patients in the control group received continuous gastrointestinal decompression, whereas patients in the treatment group received vacuum sealed drainage.

### Criteria

#### Inclusion criteria

*i*) Patients diagnosed by gastroscopy and pathological examination. *ii*) Patients who received radical gastrectomy. *iii*) Patients without metastasis.

#### Exclusion criteria

*i*) Patients who received chemotherapy and radiotherapy before surgery. *ii*) Patients diagnosed with obstruction of pylorus. *iii*) Patients who developed hypoproteinemia or anemia. *iv*) Patients who underwent total gastrectomy, bypass esophagogastrostomy or exploratory laparotomy. *v*) Patients who were extubated by accident.

### Materials

The Tape II, single-used, 1000 mL drainage bag was made by Suzhou Jingle Polymer Medical Instrument Corporation.

### Methods

The disposable gastric tubes were inserted to the patients in both groups in the morning of the surgery. When the patients returned to the ward, the disposable drainage bags attached to the negative pressure balls were connected to the gastric tubes. The bags were kept secured at the side of the bed at about 15 cm below the level of the stomach from the time the patients returned to the ward up to the time the gastric tube was removed. Continuous gastrointestinal decompression was carried out to the control group by squeezing the negative pressure ball frequently (a negative pressure of about 5.33 kPa maintained), whereas the vacuum sealed drainages of the treatment group were drained only by gravity. All other interventions conducted for both groups were the same.

### Observed index

Bowel sound recovery time, time of first flatus, indwelling time of gastric tube, and days of hospitalization were all collected from both groups. All patients were observed for postoperative complications (e.g., anastomotic leakage, intestional obstruction, wound infection, pulmonary infection, fever, and pharyngitis) throughout their hospitalization.

### Statistical analysis

All data were processed using SPSS 13.0. Measurement data were expressed as mean ± standard deviation and tested by *t*-test and ANOVA. Data were analyzed by Chi-square test, and *P*<0.05 indicated a significant difference.

## Results

### Basic information

**Table 1** shows significant differences in the bowel sound recovery time, time of the first flatus, indwelling time of gastric tube, and days of hospitalization (*P*<0.05) between the two groups. The time variables were much shorter in the treatment group than that in the control group.

**Table 1:** Comparison of time variables between the two groups.

	Treatment group (*n*=60)	Control group (*n*=60)	*T*	*P*
Time for postoperative bowel sound occurrence (h)	19.44±7.49	29.07±9.83	-2.87	0.01**
Time of the first flatus (h)	74.68±2.57	87.58±2.40	-2.29	0.03*
Indwelling gastric tube (d)	3.62±1.34	5.05±1.68	-2.60	0.01**
Days of hospitalization after the surgery (d)	13.08±1.73	14.97±2.14	-2.90	0.01**

*P<0.05,** *P*<0.01

**Table 2** shows that no statistically significant difference was found between the two groups in the occurrence of anastomotic leakage, intestinal obstruction, and wound infection (*P*>0.05). However, a significant difference was observed in the incidence of pulmonary infection, fever, and pharyngitis between the two groups (*P*<0.05), with the treatment group having a much lower incidence rate than the control group.

**Table 2:** Comparison of postoperative complications between the two groups.

Complication	Treatment group, *n* (%)	Control group, *n* (%)	χ^2^	*P*
Anastomotic leakage	1 (1.67)	2 (3.33)	0.342	0.559
Intestinal obstruction	3 (5.00)	5 (8.33)	0.536	0.464
Wound infection	2 (3.33)	4 (6.67)	0.702	0.402
Fever	4 (6.67)	9 (15.00)	4.227	0.040*
Pulmonary infection	2 (3.33)	6 (10.00)	4.821	0.028*
Pharyngitis	2 (3.33)	11 (18.33)	6.988	0.008**

*P<0.05, ** *P*<0.01

## Discussion

Continuous gastrointestinal decompression has been used in almost all patients after abdominal surgery. However, an increasing number of studies have questioned the traditional theory that supported the routine use of this method. Uninterrupted vacuum aspiration produced by continuous decompression could result in gastric mucosal hemorrhage and necrosis if the negative pressure is too strong. Moreover, continuous decompression could also lead to water-electrolyte imbalance and acid-alkali imbalance as plenty of gastric juices are drained away^[^^11^^]^. In the present study, the gastric juices of the patients in the treatment group were drained naturally by gravity; thus, incidence of gastric mucosal damage and the water-electrolyte imbalance was decreased.

Related literature has shown that some digestive juices can enter the intestines without vacuum aspiration and stimulate the function of the digestive tract, thereby contributing to the early recovery of gastrointestinal function^[^^12^^]^. In the current study, same with that of Chung’s study^[^^13^^]^, the bowel sound recovery time and the time of the first flatus were shorter in the treatment group than in the control group (*P*<0.05).

As gastric tube is painful, 97% of the patients in both groups would like to remove it earlier^[^^14^^]^. Longer indwelling time of the gastric tube may cause inflammation, ulcer, nausea, vomiting, and pulmonary infection^[^^15^^]^. Studies^[^^16^^–^^18^^]^ have reported that the infection rate among patients with gastric tube after abdominal surgery is 10 times higher than those without gastric tube. In addition, gastric tube may make the patients feel anxious and uncomfortable, which would likely affect their sleep and delay their ambulation^[^^19^^]^. The indwelling time of the gastric tubes in the treatment group was shorter than that in the control group (*P*<0.05), indicating that the gastric tube can be removed earlier in patients with vacuum sealed drainage. The incidence of complications, such as fever, pulmonary infection, and pharyngitis was lower in the treatment group than in the control group (*P*<0.05).

Li et al.^[^^20^^]^ found that continuous gastrointestinal decompression was not very useful in decreasing the pressure in the gastrointestinal tract. Studies have also found a close relationship among postoperative infection, delayed wound healing, and high intra-abdominal pressure^[^^21^^]^. However, this study found no direct relationship between the reduced gastrointestinal pressure and the complications after surgery. The incidence of anastomotic fistula, intestinal obstruction, and wound infection were the same in both groups (*P*>0.05).

Vacuum sealed drainage can accelerate the recovery of gastrointestinal function and shorten the hospitalization time of the patients, thereby decreasing medical costs and increasing the rotation rate of the hospital beds. The findings of the current study verified that continuous decompression after gastrectomy was not necessary, and the use of a vacuum sealed drainage, which has been proven to facilitate patient recovery and consequently minimize the nurses’ workload, could be an acceptable change.
